# Early Life Conditions, Adulthood Experiences, and Edentulism at Older Ages

**DOI:** 10.1093/geroni/igaa057.2900

**Published:** 2020-12-16

**Authors:** Haena Lee, Linda Waite

**Affiliations:** 1 University of Southern California, Los Angeles, California, United States; 2 University of Chicago, Chicago, Illinois, United States

## Abstract

The role of childhood in shaping overall adult health has been well documented, especially for physical and mental health, but much less is known about the impact of early disadvantage on oral health in later life. Using data from the 2006 and 2012 Health and Retirement Study, we investigate the link between childhood financial and psychosocial adversity and edentulism over a six-year period among U.S. adults aged 51 and older. We find that those growing up with parents with fewer resources face higher risks of having lost all their tooth at baseline and during the follow-up. Adulthood socioeconomic status and health behaviors are strongly associated with the risk of edentulism, net of childhood conditions. However, the effect of low parental resources on the onset of edentulism persists when accounting for these life course factors. Part of a symposium sponsored by the Oral Health Interest Group.

